# Enhancement of dynamic visual acuity using transcranial alternating current stimulation with gamma burst entrained on alpha wave troughs

**DOI:** 10.1186/s12993-023-00215-w

**Published:** 2023-08-24

**Authors:** Jimin Park, Sangjun Lee, Dasom Choi, Chang-Hwan Im

**Affiliations:** 1 Department of Electronic Engineering, Hanyang University, Seoul, Republic of Korea; 2 Department of Biomedical Engineering, Hanyang University, 222 Wangsimni-ro, Seongdong-gu, 133-791 Seoul, Republic of Korea

**Keywords:** Noninvasive brain stimulation, Transcranial alternating current stimulation (tACS), Dynamic visual acuity (DVA), Phase amplitude coupling (PAC), Inter-trial phase coherence (ITPC), Electroencephalogram (EEG)

## Abstract

**Background:**

Cross-frequency phase-amplitude coupling (PAC) of cortical oscillations is observed within and across cortical regions during higher-order cognitive processes. Particularly, the PAC of alpha and gamma waves in the occipital cortex is closely associated with visual perception. In theory, gamma oscillation is a neuronal representation of visual stimuli, which drives the duty cycle of visual perception together with alpha oscillation. Therefore, it is believed that the timing of entrainment in alpha-gamma PAC may play a critical role in the performance of visual perception. We hypothesized that transcranial alternating current stimulation (tACS) with gamma waves entrained at the troughs of alpha waves would enhance the dynamic visual acuity (DVA).

**Method:**

We attempted to modulate the performance of DVA by using tACS. The waveforms of the tACS were tailored to target PAC over the occipital cortex. The waveforms contained gamma (80 Hz) waves oscillating at either the peaks or troughs of alpha (10 Hz) waves. Participants performed computerized DVA task before, immediately after, and 10 min after each stimulation sessions. EEG and EOG were recorded during the DVA task to assess inter-trial phase coherence (ITPC), the alpha-gamma PAC at occipital site and the eye movements.

**Results:**

tACS with gamma waves entrained at alpha troughs effectively enhanced DVA, while the tACS with gamma waves entrained at alpha peaks did not affect DVA performance. Importantly, analyses of EEG and EOG showed that the enhancement of DVA performance originated solely from the neuromodulatory effects, and was not related to the modulation of saccadic eye movements. Consequently, DVA, one of the higher-order cognitive abilities, was successfully modulated using tACS with a tailored waveform.

**Conclusions:**

Our experimental results demonstrated that DVA performances were enhanced when tACS with gamma bursts entrained on alpha wave troughs were applied over the occipital cortex. Our findings suggest that using tACS with tailored waveforms, modulation of complex neuronal features could effectively enhance higher-order cognitive abilities such as DVA, which has never been modulated with conventional noninvasive brain stimulation methods.

**Supplementary Information:**

The online version contains supplementary material available at 10.1186/s12993-023-00215-w.

## Background

Theta (4-7 Hz), alpha (7-13 Hz), and gamma (>30 Hz) band activities in the occipital area have been reported to play a critical role in visual perception [1–5]. Specifically, a characteristic commonly observed in previous studies is phase-amplitude coupling (PAC) between slow (theta or alpha) and fast oscillations (gamma). In particular, alpha-gamma PAC is believed to be closely related to visual attention and cognition [1–4], whereas theta-gamma PAC is more relevant to the capacity to store visual representations [5, 6].

Gamma waves are believed to be responsible for inter-regional communication and information encoding, and alpha waves are known to rhythmically inhibit these processes [7, 8]. It is quite interesting, however, that coupling between the two waves with seemingly contradicting function plays an important role in visual perception. Theories suggest that rhythmical inhibition by alpha oscillation limits and prioritizes neuronal processing, and at the same time, a “duty cycle” is driven by gamma waves [3]. The duty cycle refers to the timing when the gamma oscillation is entrained on an oscillatory cycle of alpha waves (e.g., peak or trough) when a certain neural process occurs. Because the alpha waves serve as an inhibitory rhythm, neuronal firing is generally inhibited at the peaks of an alpha cycle, and the opposite is true at the troughs of an alpha cycle. Therefore, gamma waves cannot drive a duty cycle at the peaks of the alpha wave. In contrast, gamma waves can initiate duty cycles as inhibitory alpha waves enter their descending period. Therefore, stronger gamma waves are observed at the troughs of alpha waves [1, 7]. In summary, the entrainment timing of fast oscillating waves on slow waves is critical for neural processing in the brain.

The importance of entrainment timing in the coupling between fast and slow brain oscillations has been confirmed in a number of studies that examined information processing within a cortical region and between multiple cortical regions. For example, the performance of a visual perception task was enhanced when visual stimuli were presented over troughs of the occipital alpha [9], which was also observed in the visual cortex of monkeys [4]. Likewise, information transfer between the parietal and frontal regions was enhanced when parietal gamma waves were entrained at the troughs of the fronto-medial theta waves [10]. This evidence stresses the importance of the timing of coupling between fast and slow oscillations during higher-order cognitive processes.

Modulating the timing of gamma entrainment in slower waves over certain cortical regions could further confirm the importance of entrainment timing in neuronal processing. Such modulation could be achieved by transcranial alternating current stimulation (tACS), as tACS is known to directly influence neural activity by interacting with endogenous oscillations [11, 12] and effect large-scale cortical networks with unique resonance dynamics [13]. That is, tACS can entrain brain oscillations to specific waveforms by injecting weak alternating currents (AC) through electrodes attached over the scalp [14, 15]. Previously, only one study has focused on modulating the entrainment timing using tACS [16]. While conventional tACS employs sinusoidal AC with a single frequency [10, 17, 18], the previous study delivered a customized AC with multiple frequencies, and reported successful modulation of brain connectivity [16]. In this study, an injection current with 40 Hz gamma waves entrained over the peaks of theta waves enhanced the working memory performance. However, the injection current with 40 Hz gamma waves entrained over the troughs of theta waves did not show any significant effect. This study reiterated the role of PAC and proved that an elaborate stimulation protocol could modulate sophisticated neuronal features and eventually affect behavioral performance.

In the present study, we attempted to demonstrate that adjustment of entrainment timing in occipital alpha-gamma PAC using tACS with customized waveforms can affect the dynamic visual acuity (DVA) performance of individuals. To this end, two customized injection current waveforms were evaluated—gamma waves (80 Hz) entrained over the peaks of alpha waves (10 Hz) and gamma waves entrained over the troughs of alpha waves (hereafter referred to as the peak and trough conditions, respectively). The effect of stimulation on DVA performance was quantified by measuring DVA performance before and after both conditional and sham stimulation conditions. We hypothesized that the trough condition would strengthen the information processing of visual inputs in the occipital cortex, while the peak and sham conditions would not lead to any significant performance change. Because both image processing and visual tracking via saccadic movements are critical components of DVA [19, 20], horizontal electrooculograms (EOG) were also recorded and analyzed to observe any saccadic movement during the task. Finally, to eliminate the possibility that DVA performance was affected by the coincidental presentation of visual stimuli during certain times of occipital alpha, an electroencephalogram (EEG) was recorded from the occipital site.

## Methods

### Participants

Twenty young volunteers who had corrected to normal vision participated in the current study (ten males and ten females; mean age: 22.94 ± 2.24 years). Participants went through initial screening by identifying a gap direction of static Landolt’s C ring with gap size of 3 minimum angle of resolutions (MARs), or the smallest size of visual stimuli used in the behavioral task employed for this study. All the participants successfully identified gap directions of every static stimuli. Individuals with any identifiable neurological disorder, head injury, or personal or family history of psychiatric illness were excluded. The study protocol was approved by the Institutional Review Board (IRB) of Hanyang University, South Korea (IRB No. HYU-2020–010). The participants gave their written informed contents before the experiments. Subjects participated in three stimulation sessions, with each session (peak, trough, and sham) at least 72 h apart. No participants reported adverse symptoms, such as itchiness or phosphene.

### TACS with tailored waveform

Single-channel tACS with customized current waveforms and sham stimulation were applied to each participant on three separate days, with the current waveforms applied over the electrode at Oz. All the sessions were counterbalanced and randomized. The peak and trough sessions indicate the timing at which gamma (80 Hz) waves were entrained over the alpha (10 Hz) waves in the stimulation current waveform. The peak-to-peak amplitude of the stimulation current was 3 mA, with three cycles of 1.2 mA, 80 Hz entrained over an interval π/8, 3π/8 (peak) or interval 5π/8, 7π/8 (trough) of 1.8 mA, 10 Hz AC. For the sham condition, 10 Hz AC was ramped up to 3 mA and immediately ramped back to 0 mA once the current reached the peak, with 30 s of ramping periods. All stimulations were delivered using Starstim8 of Neuroelectrics (Barcelona, Spain).

**Fig. 1:**
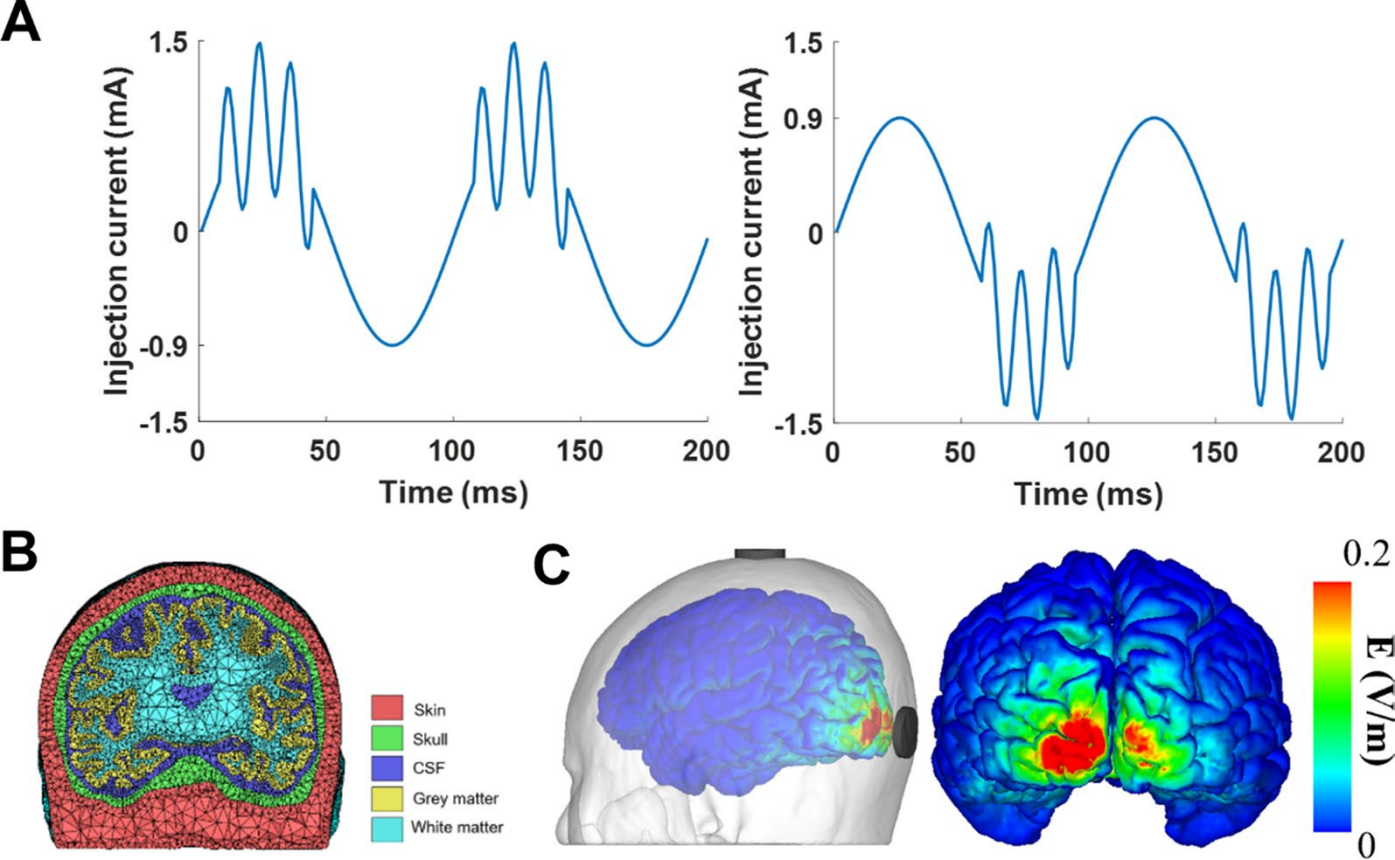
The stimulation parameters: **A** The injection current pattern, at the Oz electrode, of the peak (left) and trough (right) stimulation conditions (created using Matlab 2019b), **B** constructed finite element head model for the calculation of electric field (created using tetview), and **C** the electrode montage and electric field distribution over the cortex at phase of 0° under the stimulation setup (created using tecplot10)

The stimulation current was delivered through saline soaked, circular electrodes with an area of 25 cm^2^ placed over Cz and Oz of the 10–20 international EEG system. The stimulation montage was validated using computer simulation based on finite element method (FEM). The finite element head model was constructed from a sample MRI data (Fig. 1B), and the electric fields were computed using solver embedded in COMETS 2 [21]. The maximum electric field delivered by our setup was 0.32 V/m, which was sufficient to modulate neuronal activities [22, 23]. The electric field distribution over the cortex is illustrated in Fig. 1C. The stimulation lasted 20 min, excluding the ramping times. The ramping times were 60 s, with a ramp-up and ramp-down of 30 s each. The participants underwent three DVA task sessions: pre, post0, and post10. Pre, post0, and post10 sessions denoted the task sessions performed prior to the stimulation, immediately after the stimulation, and 10 min after the stimulation, respectively. Three participants were excluded from the analyses because they could not participate in all three stimulation (2 participants) sessions or was accidentally informed of stimulation condition (1 participant). Additionally, one participant was excluded for the analysis of 3 MARs trials since the size-specific accuracy of a participant did not reach the chance level (25%) in all the task sessions under a stimulation condition.

### Behavioral task

**Fig. 2:**
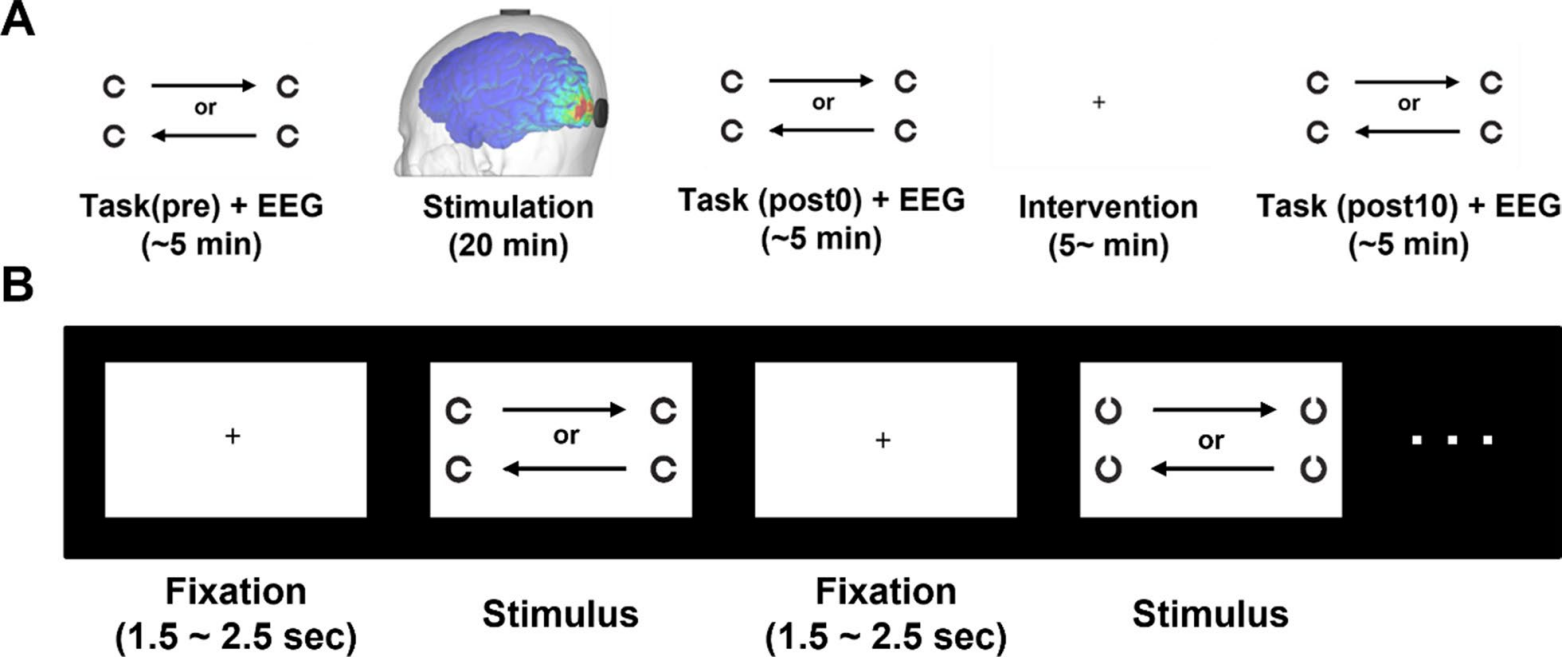
**A** The experimental protoco. The experimental protocol. Participants remained in eyes opened, resting state during intervention period, and **B** Description of a computerized DVA task session. One trial consisted of a fixation lasting 1.5–2.5 s and a Landolt’s C-ring with random gap direction (left, right, top, bottom) moving across the screen (left or right) followed by the fixation

A computerized DVA task was constructed using C# and Unity based on a previous study [20]. During the task, participants were asked to indicate the direction of the gap of a Landolt’s C-ring moving at a constant speed by using the arrow keys of the keyboard. For each trial, the stimulus presented was random in speed (from 200 to 700 degrees/s, with a step of 100 degrees/s), movement direction (left to right or vice versa), gap direction (up, down, left, and right), and size (gap size of 3, 4, and 5 MARs). All possible combinations of random factors were 144, which was the number of trials in one session. When no stimuli were presented, a fixation cross appeared on the screen. The ISI was also random, ranging from 1500 to 2500 ms. The movement of the visual stimuli covered the visual angle ranging from -10° to 10°. This range of visual angles failed to induce ocular saccades during DVA performance [20]. To maintain the visual angle, the stimuli were kept constant, and the distance between the subjects and the center of the monitor was fixed at 70 cm. To display the stimuli as fluently as possible, a 240 Hz monitor was used. A flowchart of the task session is illustrated in Fig. 2. Participants performed three sessions of computerized DVA tasks under each stimulation condition: prior to the stimulation (pre), immediately after the stimulation session (post0), and 10 min after the stimulation session (post10).

Initially, the participants underwent a screening test, wherein they were asked to identify the direction of the gap of ten 3 MARs stimuli in a static state. During the main sessions, both overall accuracy and accuracy, specific to the stimulus size, were evaluated. The experimental protocol and the schedule of tasks are illustrated in Fig. 2.

### EEG recording and analysis

During all task sessions, EEG was recorded. Of the 17 participants who completed the experiment, data from one subject were excluded from the analysis due to recording errors. The EEG data were recorded over the Oz position (the electrode at Oz site was replaced as necessary, i.e., from EEG electrode to the stimulation electrode after the pre session) according to the International 10–20 EEG system with reference attached over the right mastoid, at a sampling rate of 2,048 Hz. The recorded data were high pass filtered at 1 Hz, and 60 Hz components were notch filtered to remove power noises. In all the analyses, trials with an EEG amplitude higher than 100 µV were excluded (maximum of 14 trials were removed, from one subject in post0 session of peak stimulation condition) from the analysis to eliminate trials contaminated with noise. For trials that were not contaminated, the inter-trial phase coherence (ITPC) and power spectrum was computed and averaged across trials for each participant. The ITPC was computed using *newtimf* function of EEGLAB in MATLAB version 2019b [24], with frequency range of 1 to 100 Hz with a step size of 1 Hz. The power spectrum was computed based on *fft* function in MATLAB. Finally, the PAC was computed using a MATLAB toolbox available at https://data.mrc.ox.ac.uk; the PAC was computed for phase frequency of 4 Hz to 20 Hz with a step size of 1 Hz and amplitude frequency of 30 Hz to 100 Hz with a step size of 5 Hz. All the features were computed for the time window spanning -1000 ms to 0 ms, where 0 ms was the stimulus onset.

### Statistical analysis

All the statistical analyses were conducted using Matlab 2019b. For the behavioral data, both the qq-plot and data distribution indicated that the data did not follow normal distribution, as confirmed by the Shapiro–Wilk test (see Supplementary information for details). Therefore, a nonparametric analysis was employed. For behavioral performance data, Friedman’s test for within factor ‘session’ was performed to examine the overall and size-specific accuracy under each stimulation condition. Additionally, Friedman’s test was performed for within factor ‘Stimulation condition’ to assess the overall and size-specific accuracy of pre sessions to evaluate whether there was any difference between the baseline conditions for each day. Then, a post-hoc analysis of Wilcoxon’s signed-rank test was performed if necessary. Finally, multiple corrections of *p*-values were made using false discovery rate (FDR).

For EEG data, statistical analyses were conducted for the ITPC, 10 Hz power, 80 Hz power, and PAC. For every feature, Friedman’s test was performed for within factor ‘session’, and subsequent Wilcoxon’s signed rank test was performed if necessary. All the *p*-values acquired from the Wilcoxon’s signed rank test was corrected using FDR. Because stimulation parameters targeted alpha (10 Hz) and high gamma (80 Hz), appropriate windows of interests were selected for each analysis.

For ITPC, six windows of interests for the statistical analyses were selected: all possible combinations between two frequency windows of 7 − 13 Hz and 70 − 90 Hz and 200 ms time windows starting from − 600 ms, − 400 ms, and − 200 ms. The time windows were chosen to represent time windows prior to the visual stimuli onset, as phase alignment during visual perception is reported to occur prior to the stimulus onset. For PAC, the statistical analyses were conducted for a phase frequency bin of 7 − 13 Hz and amplitude frequency bin of 70 − 90 Hz. The mean PAC value for the window of interest was computed for each session under the stimulation conditions, and Friedman’s test was performed. Since Friedman’s test did not yield any significance, no post-hoc analysis was conducted.

## Results

### Computer simulation

The simulation results revealed that the maximum electric field amplitude induced over the cortex was 0.32 V/m when 1.5 mA was applied over the Oz electrode (Fig. 1C), which is strong enough to entrain neuronal activities [25–27]. Note that the simulation was conducted assuming that maximal current was applied over the Oz electrode.

### Behavioral performance

**Fig. 3:**
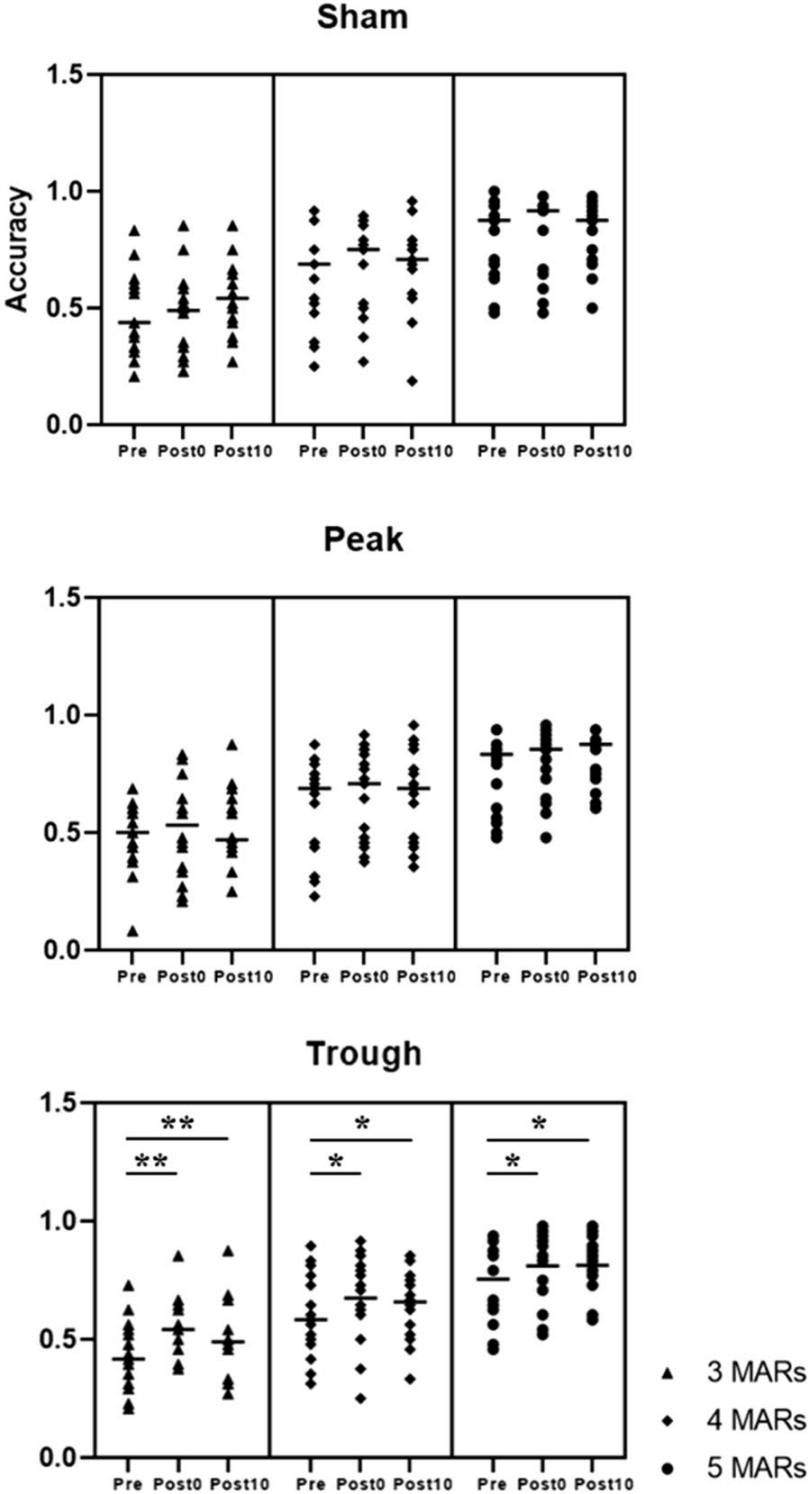
Behavioral performance of 3, 4, and 5 MARs stimuli under each stimulation condition. Only the trough condition exhibited a significant increase in accuracy after the stimulation compared to the baseline performance (created using graphpad prism 7)

Because the data did not follow normal distribution, nonparametric methods were used for all statistical analyses. Exemplary quantile–quantile plot and distribution of the data are illustrated in Supplementary information (Additional file 1: Fig. S1). More detailed information about the task is described in the Methods section and Fig. 2. Friedman’s tests with within-factor ‘sessions’ revealed significant differences in all size-specific and overall accuracies under trough conditions (3 MARs: χ^2^ = 10.32, p < 0.01; 4 MARs: χ^2^ = 7.85, p = 0.02; 5 MARs: χ^2^ = 8.39, p = 0.015; overall: χ^2^ = 13, *p* < 0.01) and the accuracy of 5 MARs stimuli under the sham condition (χ^2^ = 6.64, *p* = 0.04). Additionally, the post-hoc analysis revealed significant differences between the accuracies of pre-post0 and pre-post10 only under the trough condition for all the measures (between pre-post0: *p* < 0.01 for 3 MARs, 4 MARs, and the overall accuracy, and *p* = 0.026 for 5 MARs; between pre-post10: *p* < 0.01 for 3 MARs, 4 MARs, and the overall accuracy, and *p* = 0.011 for 5 MARs). However, no significant difference was observed between the accuracies of 5 MARs under the sham session (p > 0.14). In addition, there were no significant differences between the accuracies of the different pre-sessions (3 MARs: χ^2^ = 1.66, *p* = 0.44; 4 MARs: χ^2^ = 3.31, *p* = 0.19; 5 MARs: χ^2^ = 1.61, *p* = 0.45; overall: χ^2^ = 2.38, *p* = 0.3). These results indicate that there was no difference in the participants’ baseline performance, regardless of the stimulation conditions. All p-values presented were corrected using the FDR. The behavioral results are shown in Fig. 3.

### EEG analysis

**Fig. 4:**
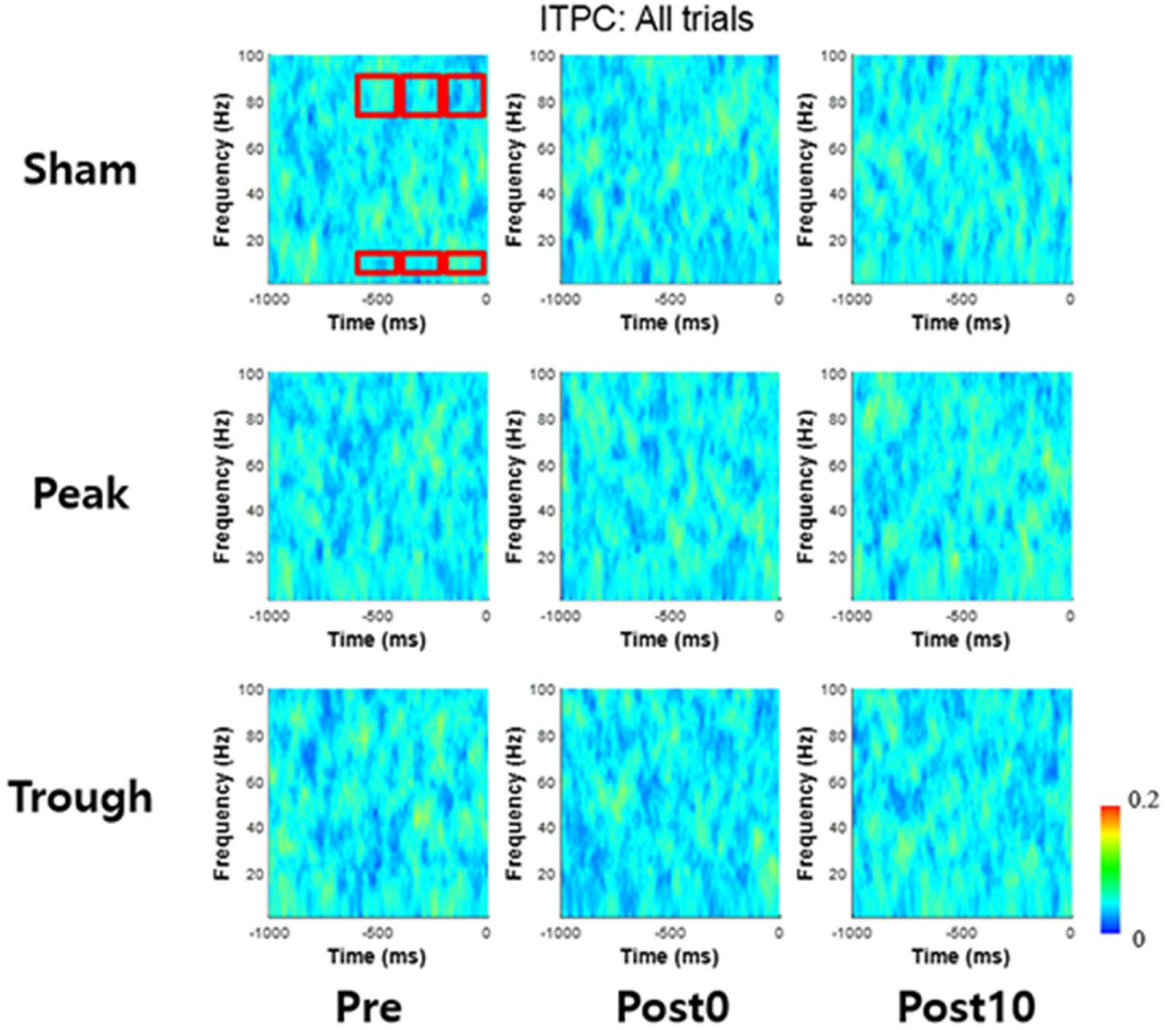
The inter trial phase coherence from -1000 ms to 0 ms from stimulus onset. Each row (from top to bottom) represents stimulation condition of sham, peak, and trough. Each column (from left to right) represents sessions of pre, post0, and post10. The red squares indicate the windows of interests (created using Matlab 2019b)

EEG was recorded during each task session. EEG was recorded at Oz, with the reference attached over right mastoid. To examine whether the changes in behavioral performance was due to the previously reported pre-stimulus phase realignment, ITPC was computed (Fig. 4). For the windows of interest, no significant difference was observed for post0 and post10 sessions compared to pre session, regardless of the stimulation conditions (χ^2^ < 5.38, *p* > 0.068 for each comparison, per Friedman’s test, where the complete statistics are displayed in Table. 1). The windows of interests covered time windows prior to the stimulus onset, as significant phase alignment is usually observed prior to the stimulus onset during visual perception [28]. This implies that the behavioral changes were not caused by coincidental alignment of pre-stimulus phase. Furthermore, the ITPC was computed separately for correct and missed trials, as the significant phase alignment was better observed in correct trials [9]. However, significant increase in phase alignment due to different stimulation conditions were not observed for both correct trials and missed trials (depicted in Additional file 1: Fig. S2).

**Table 1:** Friedman’s test for ITPC for each window of interest under stimulation conditions

	Sham	Peak	Trough
− 600 to − 400 ms, 7 to 13 Hz	χ^2^ = 0.88, *p* = 0.64	χ^2^ = 0.88, *p* = 0.64	χ^2^ = 4.88, *p* = 0.09
− 400 to − 200 ms, 7 to 13 Hz	χ^2^ = 2.63, *p* = 0.27	χ^2^ = 1.63, *p* = 0.44	χ^2^ = 4.88, *p* = 0.09
− 200 to 0 ms, 7 to 13 Hz	χ^2^ = 0.88, *p* = 0.64	χ^2^ = 2.38, *p* = 0.31	χ^2^ = 0.13, *p* = 0.94
− 600 to − 400 ms, 70 to 90 Hz	χ^2^ = 3.5, *p* = 0.17	χ^2^ = 4.63, *p* = 0.1	χ^2^ = 1.63, *p* = 0.44
− 400 to − 200 ms, 70 to 90 Hz	χ^2^ = 4.63, *p* = 0.1	χ^2^ = 1.63, *p* = 0.44	χ^2^ = 1.44, *p* = 0.14
− 200 to 0 ms, 70 to 90 Hz	χ^2^ = 0.38, *p* = 0.83	χ^2^ = 5.38, *p* = 0.068	χ^2^ = 1.63, *p* = 0.44

**Fig. 5:**
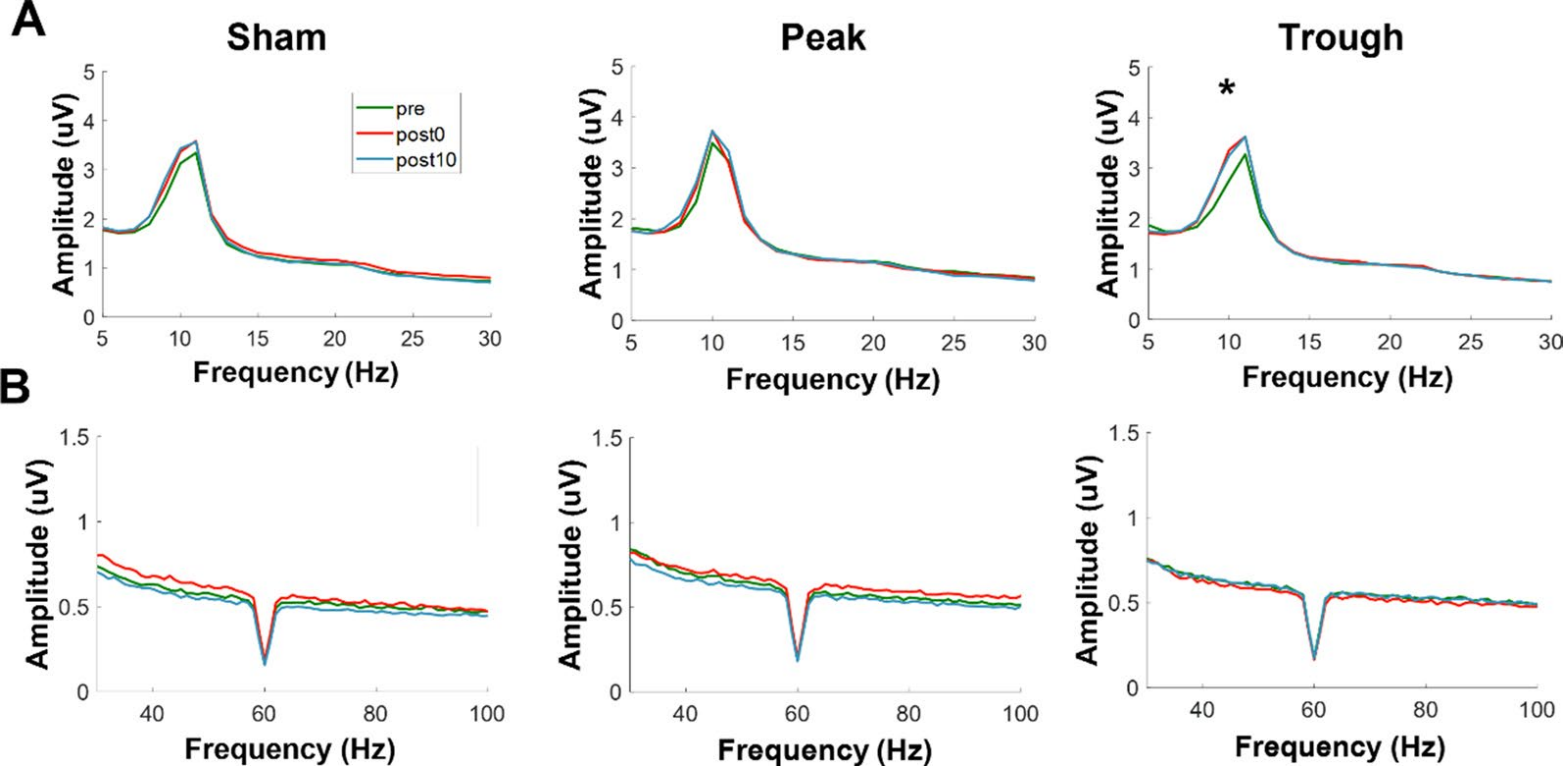
The power spectra **A** from 5 to 30 Hz and **B** from 30 to 100 Hz. Each column, from left to right, of the power spectra indicates stimulation conditions of sham, peak, and trough, respectively. The green, red, and blue lines indicate power spectra during pre, post0, and post10 sessions, respectively (created using Matlab 2019b)

To further test the effect of stimulation conditions, the additional features during the task was observed: i) the power spectrum of 10 Hz and 80 Hz and ii) the PAC were computed. Firstly, the spectral power of 10 Hz significantly increased only after the trough stimulation condition, in both post0 and post10 sessions compared to the pre session (Fig. 5A-B). For clarity of display, error bars were omitted; the power spectrum with standard error shaded is illustrated in (Additional file 1: Fig. S3). While Friedman test for within factor ‘session’ showed significant difference between sessions for each of stimulation conditions (sham: χ^2^ = 6, *p* = 0.05; peak: χ^2^ = 6.5, *p* = 0.039; trough: χ^2^ = 12.13, *p* < 0.01), subsequent post-hoc analysis of Wilcoxon’s signed rank test with multiple correction using FDR showed significance only under the trough stimulation condition (between pre-post0 and pre-post10, respectively, sham: *p* = 0.2 and *p* = 0.17, peak: *p* = 0.21 and *p* = 0.08, and trough: *p* < 0.01 and *p* = 0.011). No significance was found between post0 and post10 sessions, regardless of stimulation conditions (*p* > 0.19). All the *p*-values reported are corrected using FDR. Additionally, no significance was found for 80 Hz in Friedman test, albeit marginal significance was observed for the trough condition (sham: sham: χ^2^ = 0.38, *p* = 0.83; peak: χ^2^ = 2, *p* = 0.37; trough: χ^2^ = 4.88, *p* = 0.09).

**Fig. 6:**
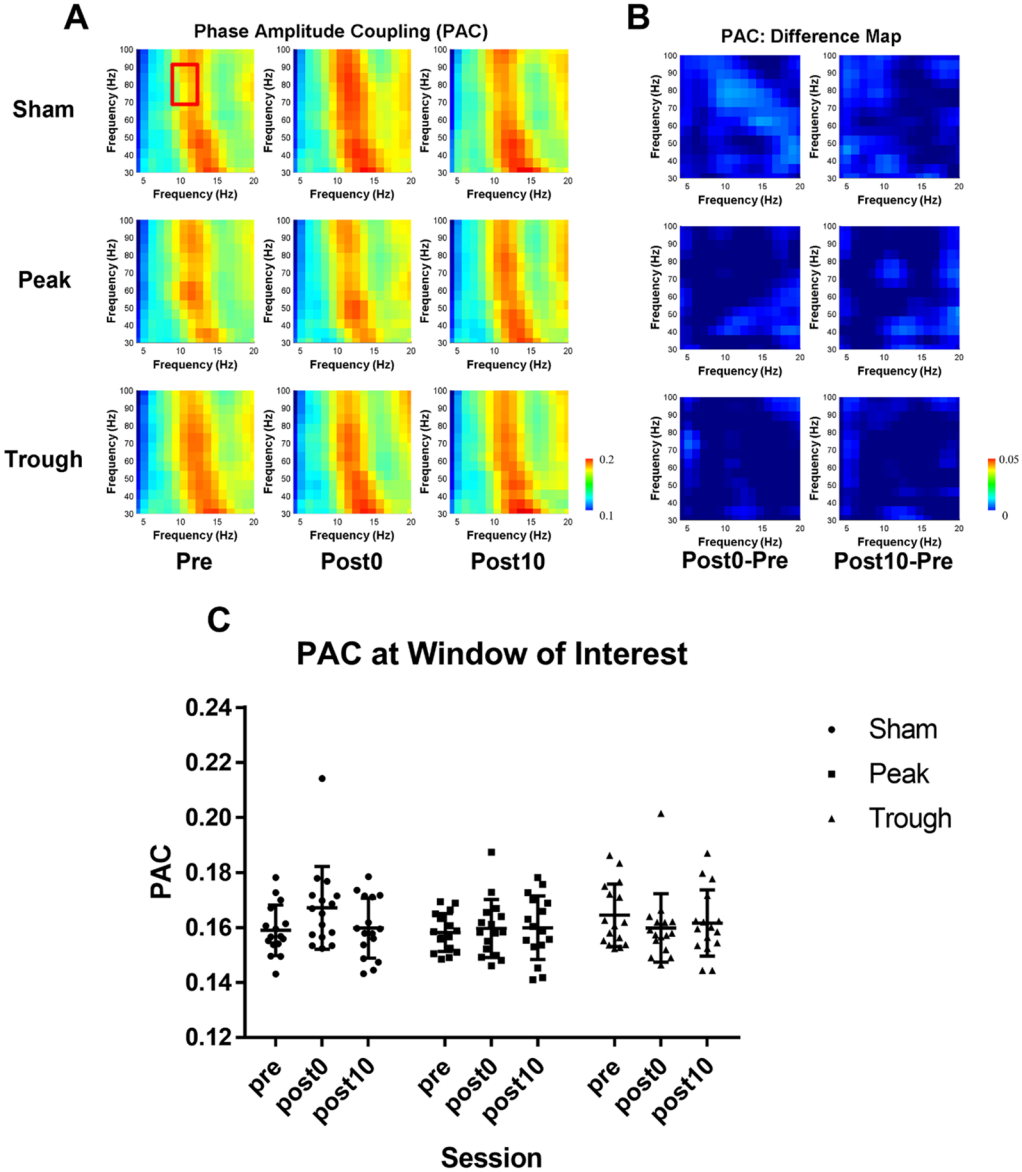
**A** The spectrogram of PAC for each of the completed nine sessions; The red square indicates the window of interest (created using Matlab 2019b), **B** difference between PAC of post0/post10 session and pre session, from left to right (created using Matlab 2019b), and **C** the distribution of average PAC at the window of interest (created using graphpad prism 7)

Secondly, as in Fig. 6, relatively strong PAC between alpha and high gamma was observed throughout the behavioral performance. However, Friedman’s test for factor ‘session’ showed no significant difference between PAC of alpha (7 ~ 13 Hz) and high gamma (70 ~ 90 Hz) during post0 and post10 sessions compared to the pre sessions (sham: χ^2^ = 4.63, *p* = 0.1; peak: χ^2^ = 0.13, *p* = 0.94; trough: χ^2^ = 5.38, *p* = 0.07). The PAC results indicate that while alpha-high gamma coupling is related to behavioral performance, stimulations did not alter alpha-high gamma PAC at the occipital site. This result is in line with that of spectral power analysis, as amplitudes of gamma were not affected by stimulation.

## Discussion

In this study, we sought to improve DVA performance using tACS with customized AC waveforms and found that the modulation of spatiotemporal features of alpha-gamma PAC at the occipital site could successfully enhance DVA performance. It is widely believed that visual perception is controlled by the inhibitory cycle of occipital alpha waves [29–32]. Specifically, visual inputs are most efficiently processed during the descending period, or troughs, of alpha waves [1, 7]. It could be argued that to assess such behavioral performance related to visual perception, the employment of static visual stimuli might be sufficient rather than presenting a dynamic visual stimulus; however, improving the identification of static visual stimuli would be related more to vision itself than to visual processing mechanisms related to occipital cortex.

The main finding of our stimulation study was in agreement with previous studies, as DVA performance was enhanced only when gamma oscillations were entrained on the troughs of the alpha waves. For the trough condition, 80 Hz gamma waves were entrained over the [17π/16, 31π/16] period of the 10 Hz alpha phase, whereas the gamma waves were entrained over the [π/16, 15π/16] period for the peak condition. We chose 10 Hz and 80 Hz as the stimulation parameters because 10 Hz is a widely used frequency when targeting alpha oscillations [33, 34] and it is reported that high gamma (> 50 Hz) being coupled with alpha at the posterior cortex is critical during visual tasks [35]. It could be debated whether weak AC delivered transcranially can modulate such intricate cortical rhythms [36]. However, an ephaptic coupling hypothesis [37] and many other reports [15, 38–40] underpin the feasibility of such modulation. Therefore, we believe that the improvement in DVA performance in our study originated from the modulation of entrainment timing in the alpha-gamma PAC at the occipital site.

Since previous studies reported prestimulus oscillatory phase affecting visual perceptions, we set the inter-stimulus interval (ISI) as a random value between 1.5 and 2.5 s in an effort to randomize the prestimulus oscillatory phases. Furthermore, we recorded EEG signals at Oz during the task sessions and analyzed the ITPC from – 1000ms to 0 ms from stimulus onset. ITPC were not affected by stimulation, as evidenced by the lack of a significant difference between sessions after and prior to each stimulation. Therefore, it was confirmed that the visual stimuli of the DVA task was not presented at specific oscillatory phases. This further supports that the observed enhancement of DVA performance was not a product of coincidental alignment of the visual stimuli to a certain phase of the occipital alpha.

Although the stimulation parameters included both alpha (10 Hz) and gamma (80 Hz) components, the power spectrum showed that only the power of 10 Hz was enhanced when trough stimulation was applied, while the 80 Hz component was not significantly affected by stimulations. Therefore, it could be argued that only applying 10 Hz stimulation might be sufficient to target causal oscillatory features for DVA performance. However, we firmly believe that this argument is not true based on the following two reasons: Firstly, the peak stimulation condition did not enhance either DVA performance or 10 Hz power, although the peak stimulation parameter also included 10 Hz. Secondly, the previous literature that employed a similar tACS waveform yielded similar behavioral and EEG results to ours, where behavioral performances were dependent on the frequency of the higher frequency component in the stimulation waveform while EEG functional connectivity for the low frequency component over cortical regions was increased only when the stimulation was effective [16]. Note that our stimulation amplitude ratio between the phase and amplitude frequencies (10 Hz and 80 Hz, respectively) was replicated from the study.

To the best of our knowledge, no previous study has explored the causality between the different amplitudes of the phase and amplitude frequencies and cognitive measures. Although, given that the brain oscillations naturally exhibit higher power in the low frequencies (i.e., alpha range, 7–13 Hz) than high frequencies (i.e., gamma range, 30–100 Hz), it seems feasible that the low frequency component (10 Hz) in our stimulation waveform has higher amplitude than the high frequency component (80 Hz). Granted, the optimal ratio or amplitude of each frequency component when designing stimulation waveform likely depends on individual responsiveness to stimulations. Such individualization of stimulation amplitude is extremely challenging to achieve in most experimental designs, including the current study. Furthermore, we aimed to modulate frequency coupling in a single cortical region by manipulating temporal dynamics of the tACS waveforms. Combined, while the amplitudes of each frequency component constituting the customized tACS waveform might be important, a focus should be placed on the temporal characteristics and amplitude of the entire waveform when evaluating the waveform.

In an effort to examine whether the increase in alpha power truly affected behavioral performance, we also computed event-related spectral perturbation (ERSP) during the task. The results indicated that the trough stimulation condition significantly increased ERSP at the alpha band prior to the onset of visual stimuli, further underpinning the trough stimulation condition only affected alpha band (see Additional file 1: Fig. S4 for details).

There are two key components of the DVA: visual information processing and visual search through saccadic movements. Several previous studies that compared DVA performance between normal people and athletes reported that faster saccadic movements generally result in better DVA performance [19, 20]. To further validate whether the tACS with a tailored waveform was the only factor involved in the enhancement of DVA performance, we observed saccadic movements from EOG signals recorded during the entire task. Since we recorded EOG only during the task, we were unable to statistically claim that there was no eye movement. However, saccadic movements are rapid and thus could be easily detected by visual inspection. In this light, no significant saccadic movement was observed during any task sessions (Additional file 1: Fig. S5), indicating that the DVA performance was unaffected by saccadic movement changes.

The causal role of brain oscillations in cognitive performance has been widely studied [41–43]. Specifically, our results are in agreement with those of previous studies that emphasized the close relationship between the entrainment timing of cross-frequency PAC and behavioral performance [4, 9, 10]. In the present study, we demonstrated that modulation of the entrainment timing of alpha-gamma PAC using tACS with a tailored waveform could successfully alter visual processing performance, particularly the DVA performance. Nevertheless, we did not conduct any neuroimaging study that might quantitatively evaluate the brain network dynamics, especially the fronto-occipital network [44], altered by tACS. Further exploration of the causality between brain network dynamics and the modulation of entrainment timing in PAC should be performed using neuroimaging methods.

## Conclusion

This study attempted to modulate the entrainment timing of gamma oscillations at alpha oscillations using tACS with tailored waveforms. Our experimental results demonstrated that tACS over the occipital cortex with gamma bursts entrained on alpha wave troughs could effectively enhance DVA. Our findings suggest that using tACS with tailored waveforms, modulation of complex neuronal features could effectively enhance higher-order cognitive abilities such as DVA, which has never been modulated with conventional noninvasive brain stimulation methods. It is expected that our findings may provide useful insights for developing new interventions based on noninvasive brain stimulation.

### Supplementary Information

**Additional file 1: ** Additional analyses of data.

## Data Availability

The measured data and code can be conditionally accessed upon request to the corresponding author due to privacy and conditions of approvement by the Institutional Review Board of Hanyang University.

## Abbreviations

PAC: Phase amplitude coupling
tACS: Transcranial alternating current stimulation
NIBS: Noninvasive brain stimulation
AC: Alternating current
DVA: Dynamic visual acuity
EOG: Electrooculogram
EEG: Electroencephalogram
MAR: Minimum angle of resolution
FEM: Finite element method
ITPC: Inter-trial phase coherence
FDR: False discovery rate
ERSP: Event-related spectral perturbation

## References

[1] VanRullen R (2016). Perceptual cycles. Trends Cogn Sci.

[2] Tzvi E, Bauhaus LJ, Kessler TU, Liebrand M, Wöstmann M, Krämer UM (2018). Alpha-gamma phase amplitude coupling subserves information transfer during perceptual sequence learning. Neurobiol Learn Mem.

[3] Jensen O, Gips B, Bergmann TO, Bonnefond M (2014). Temporal coding organized by coupled alpha and gamma oscillations prioritize visual processing. Trends Neurosci.

[4] Spaak E, Bonnefond M, Maier A, Leopold DA, Jensen O (2012). Layer-specific entrainment of gamma-band neural activity by the alpha rhythm in monkey visual cortex. Curr Biol.

[5] Demiralp T, Bayraktaroglu Z, Lenz D, Junge S, Busch NA, Maess B (2007). Gamma amplitudes are coupled to theta phase in human EEG during visual perception. Int J Psychophysiol.

[6] Skaggs WE, McNaughton BL, Wilson MA, Barnes CA (1996). Theta phase precession in hippocampal neuronal populations and the compression of temporal sequences. Hippocampus.

[7] Schroeder CE, Lakatos P (2009). The gamma oscillation: master or slave?. Brain Topogr.

[8] Fukai T (2000). Neuronal communication within synchronous gamma oscillations. NeuroReport.

[9] Hanslmayr S, Volberg G, Wimber M, Dalal SS, Greenlee MW (2013). Prestimulus oscillatory phase at 7 Hz gates cortical information flow and visual perception. Curr Biol.

[10] Pahor A, Jaušovec N (2014). The effects of theta transcranial alternating current stimulation (tACS) on fluid intelligence. Int J Psychophysiol.

[11] Krause MR, Vieira PG, Thivierge J-P, Pack CC (2022). Brain stimulation competes with ongoing oscillations for control of spike timing in the primate brain. PLoS Biol.

[12] Vieira PG, Krause MR, Pack CC (2020). tACS entrains neural activity while somatosensory input is blocked. Plos Biol.

[13] Ali MM, Sellers KK, Fröhlich F (2013). Transcranial alternating current stimulation modulates large-scale cortical network activity by network resonance. J Neurosci.

[14] Antal A, Paulus W (2013). Transcranial alternating current stimulation (tACS). Front Hum Neurosci.

[15] Helfrich RF, Schneider TR, Rach S, Trautmann-Lengsfeld SA, Engel AK, Herrmann CS (2014). Entrainment of brain oscillations by transcranial alternating current stimulation. Curr Biol.

[16] Alekseichuk I, Turi Z, de Lara GA, Antal A, Paulus W (2016). Spatial working memory in humans depends on theta and high gamma synchronization in the prefrontal cortex. Curr Biol.

[17] Pollok B, Boysen A-C, Krause V (2015). The effect of transcranial alternating current stimulation (tACS) at alpha and beta frequency on motor learning. Behav Brain Res.

[18] Wolinski N, Cooper NR, Sauseng P, Romei V (2018). The speed of parietal theta frequency drives visuospatial working memory capacity. PLoS Biol.

[19] Uchida Y, Kudoh D, Higuchi T, Honda M, Kanosue K (2013). Dynamic visual acuity in baseball players is due to superior tracking abilities. Med Sci Sports Exerc.

[20] Uchida Y, Kudoh D, Murakami A, Honda M, Kitazawa S (2012). Origins of superior dynamic visual acuity in baseball players: superior eye movements or superior image processing. PLoS ONE.

[21] Lee C, Jung Y-J, Lee SJ, Im C-H (2017). COMETS2: an advanced MATLAB toolbox for the numerical analysis of electric fields generated by transcranial direct current stimulation. J Neurosci Methods.

[22] Reato D, Rahman A, Bikson M, Parra LC (2010). Low-intensity electrical stimulation affects network dynamics by modulating population rate and spike timing. J Neurosci.

[23] Herrmann CS, Rach S, Neuling T, Strüber D (2013). Transcranial alternating current stimulation: a review of the underlying mechanisms and modulation of cognitive processes. Front Hum Neurosci.

[24] Makeig S, Debener S, Onton J, Delorme A (2004). Mining event-related brain dynamics. Trends Cogn Sci.

[25] Kantrowitz JT, Sehatpour P, Avissar M, Horga G, Gwak A, Hoptman MJ (2019). Significant improvement in treatment resistant auditory verbal hallucinations after 5 days of double-blind, randomized, sham controlled, fronto-temporal, transcranial direct current stimulation (tDCS): a replication/extension study. Brain Stimul.

[26] Vöröslakos M, Takeuchi Y, Brinyiczki K, Zombori T, Oliva A, Fernández-Ruiz A (2018). Direct effects of transcranial electric stimulation on brain circuits in rats and humans. Nat Commun.

[27] Lee WH, Kennedy NI, Bikson M, Frangou S (2018). A computational assessment of target engagement in the treatment of auditory hallucinations with transcranial direct current stimulation. Front Psych.

[28] Hülsdünker T, Strüder HK, Mierau A (2018). The pre-stimulus oscillatory alpha phase affects neural correlates of early visual perception. Neurosci Lett.

[29] Van Dijk H, Schoffelen J-M, Oostenveld R, Jensen O (2008). Prestimulus oscillatory activity in the alpha band predicts visual discrimination ability. J Neurosci.

[30] Mathewson KE, Lleras A, Beck DM, Fabiani M, Ro T, Gratton G (2011). Pulsed out of awareness: EEG alpha oscillations represent a pulsed-inhibition of ongoing cortical processing. Front Psychol.

[31] Kizuk SA, Mathewson KE (2017). Power and phase of alpha oscillations reveal an interaction between spatial and temporal visual attention. J Cogn Neurosci.

[32] Landau AN, Fries P (2012). Attention samples stimuli rhythmically. Curr Biol.

[33] Moliadze V, Sierau L, Lyzhko E, Stenner T, Werchowski M, Siniatchkin M (2019). After-effects of 10 Hz tACS over the prefrontal cortex on phonological word decisions. Brain Stimul.

[34] Wach C, Krause V, Moliadze V, Paulus W, Schnitzler A, Pollok B (2013). The effect of 10 Hz transcranial alternating current stimulation (tACS) on corticomuscular coherence. Front Hum Neurosci.

[35] Voytek B, Canolty RT, Shestyuk A, Crone NE, Parvizi J, Knight RT (2010). Shifts in gamma phase–amplitude coupling frequency from theta to alpha over posterior cortex during visual tasks. Front Hum Neurosci.

[36] Buzsáki G, Anastassiou CA, Koch C (2012). The origin of extracellular fields and currents—EEG, ECoG. LFP and spikes Nature Reviews Neuroscience.

[37] Qiu C, Shivacharan RS, Zhang M, Durand DM (2015). Can neural activity propagate by endogenous electrical field?. J Neurosci.

[38] Jaušovec N, Jaušovec K (2014). Increasing working memory capacity with theta transcranial alternating current stimulation (tACS). Biol Psychol.

[39] Kasten FH, Herrmann CS (2017). Transcranial alternating current stimulation (tACS) enhances mental rotation performance during and after stimulation. Front Hum Neurosci.

[40] Kanai R, Paulus W, Walsh V (2010). Transcranial alternating current stimulation (tACS) modulates cortical excitability as assessed by TMS-induced phosphene thresholds. Clin Neurophysiol.

[41] Riddle J, McFerren A, Frohlich F (2021). Causal role of cross-frequency coupling in distinct components of cognitive control. Prog Neurobiol.

[42] Beliaeva V, Savvateev I, Zerbi V, Polania R (2021). Toward integrative approaches to study the causal role of neural oscillations via transcranial electrical stimulation. Nat Commun.

[43] Zhang Y, Zhang Y, Cai P, Luo H, Fang F (2019). The causal role of α-oscillations in feature binding. Proc Natl Acad Sci.

[44] Hamm JP, Dyckman KA, McDowell JE, Clementz BA (2012). Pre-cue fronto-occipital alpha phase and distributed cortical oscillations predict failures of cognitive control. J Neurosci.

