# Supplementary material for: Enhancement of dynamic visual acuity using transcranial alternating current stimulation with gamma burst entrained on alpha wave troughs
Source: Behav Brain Funct. 2023 Aug 24;19:13. doi: 10.1186/s12993-023-00215-w (PMC10463531; doi:10.1186/s12993-023-00215-w)
Supplement: Supplementary file 1 — Additional file 1: Additional analyses of data. [file 12993_2023_215_MOESM1_ESM.pdf]

Supplementary Information

Full Title:

Enhancement of Dynamic Visual Acuity Using Transcranial Alternating Current Stimulation with Gamma Burst Entrained on Alpha Wave Troughs

Jimin Park[1], Sangjun Lee[1], Dasom Choi[1] and Chang-Hwan Im[1, 2*¶]

[1] Department of electronic Engineering, Hanyang University, Seoul, Republic of Korea

[2] Department of biomedical Engineering, Hanyang University, Seoul, Republic of Korea

1. The distribution of behavioral data

Because the number of participants were small, the quantile-quantile plot (qq-plot) and distribution were drawn from the behavioral data. Exemplary qq-plot and the distribution of the data with curve fitted to the closest normal distribution is depicted using post0 session under peak stimulation condition (Fig. S1).

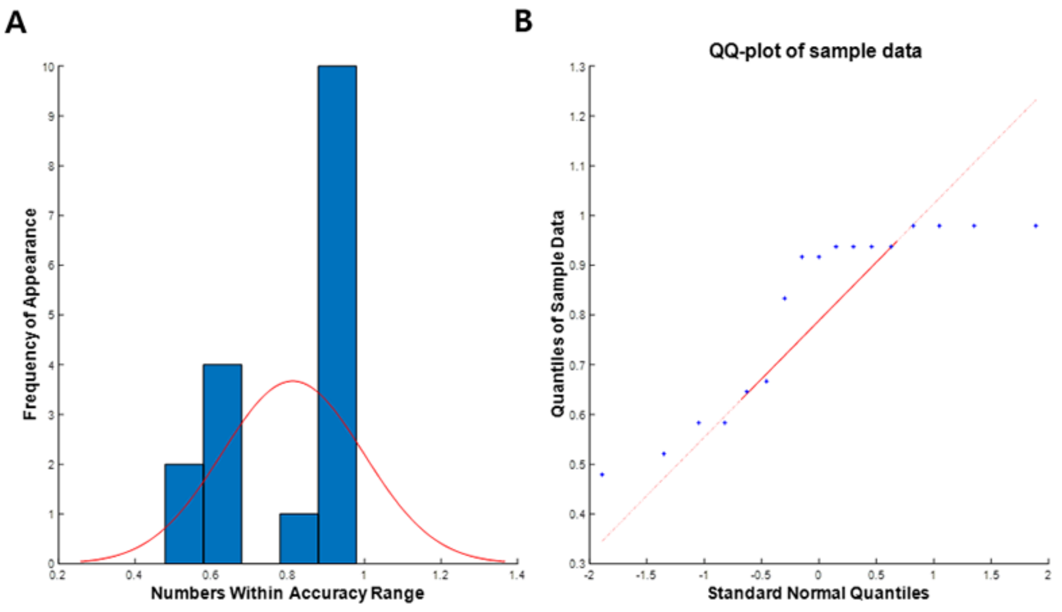

Figure S1. (a) The histogram of accuracy of 5 MARS stimuli when performing post0 session under sham condition, (b) the quantile-quantile plot of the sample data

2. Inter-trial phase coherence (ITPC)

It was reported that the phase alignment prior to the visual stimulus onset was better observed in correct trials. Thus, we tested whether our stimulation conditions specifically affected phase alignment of either correct or missed trials by computing ITPC separately (Fig. S2). Overall, while ITPC was higher in missed trials compared to correct trials, no significant difference was observed for either correct or missed trials for any frequency component at any timepoint. Because there are no trends observed in missed trials, overall ITPC value being high in missed trial seems to be effect of number of missed trials being significantly smaller than the correct trials, as ITPC is a measure significantly influenced by number of trials assessed.

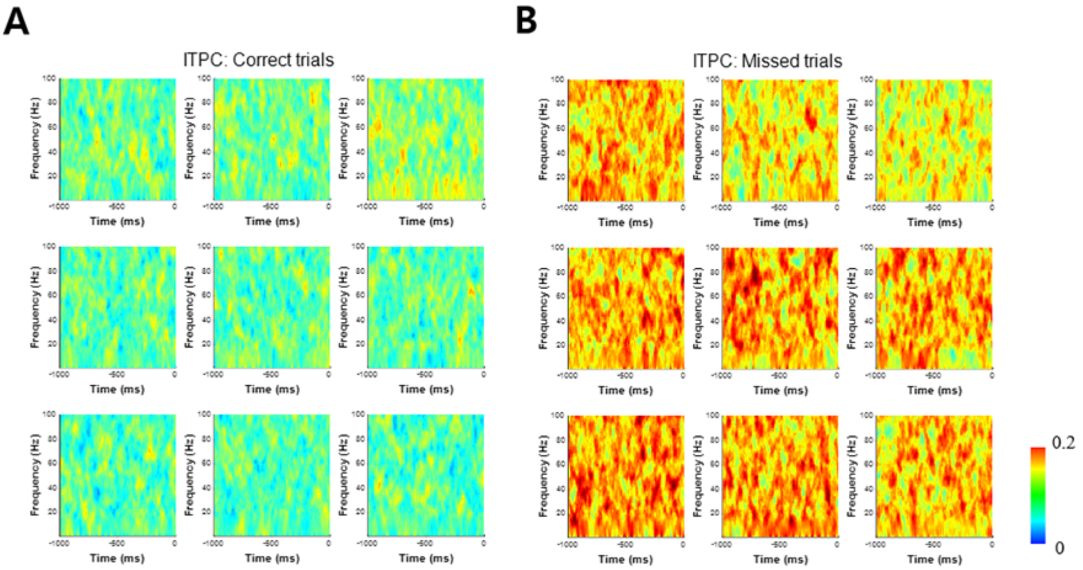

Figure S2. The ITPC distribution during -1000 ms to 0 ms, with time relative to the visual stimulus onset, of (a) correct trials and (b) missed trials.

3. The frequency power spectrum

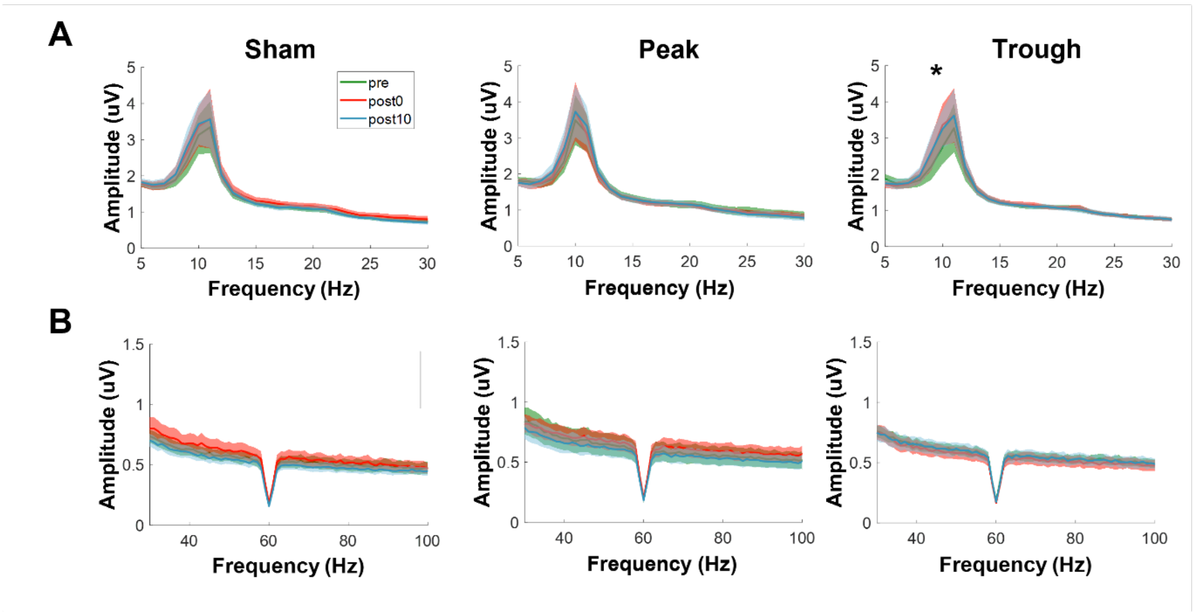

Figure S3. The frequency power spectrum for (a) alpha and beta range and (b) gamma range. Each column from left to right indicates the stimulation condition of sham, peak, and trough, respectively. The shaded areas indicate standard error.

4. Event-related spectral perturbation (ERSP)

Using the EEG signal from -1000 ms to 500 ms since the stimulus onset, ERSP was computed using eeglab and in-house MATLAB code (Fig. S3). Signal from -1000 ms to -800 ms was used as baseline. Since the inter-stimulus interval (ISI) was jittered from 1500 ms to 2500 ms, a 1500 ms window was the longest time frame without signal for each trial overlapping each other. Statistical analyses were performed for the same windows of interests as ITPC (six total windows from combination between two frequency windows of 7 to 13 Hz and 70 to 90 Hz and 200 ms time windows starting at -600 ms, -400 ms, and -200 ms. Friedman's test using within factor 'sessions' showed significant difference during peak condition (-400 ms to -200 ms, alpha band: $\chi^2 = 6.13$, $p = 0.046$) and alpha band windows of trough condition (-600 ms to -400ms: $\chi^2 = 7.63$, $p = 0.02$; -400 ms to -200ms: $\chi^2 = 9.5$, $p = 0.008$; -200 ms ~ 0ms: $\chi^2 = 9.5$, $p = 0.008$). Subsequent Wilcoxon's signed rank test showed that ERSP were higher during post0 session compared to pre session for significant window of peak condition (pre-post0: $p = 0.02$, pre-post10: $p = 0.32$, post0-post10: $p = 0.06$). This was true for all the significant windows under the trough condition, with ERSP during post0 session also being higher than post10 session for time window of -200 ms to 0 ms. The *p*-values for each time window under the trough condition is described in Table S1. We initially hypothesized that gamma entrained may increase event-related activity rather than increasing amplitude of gamma power (as was in previous study), lack of increase in ERSP during post10 session leaves open to discussion whether the ERSP is actually a feature correlated to DVA performance. However, it seems obvious that stimulation, especially trough condition, affected event-related activities possibly related to more than just low frequency oscillation.

Table S1. ***p*-values for windows of interests with significance, under trough condition**

	Pre-post0	Pre-post10	Post0-post10
-600 ms to –400 ms	**0.03**	0.5	0.09
-400 ms to –200 ms	**0.02**	0.6	0.09
-200 ms to 0 ms	< **0.01**	0.44	**0.045**

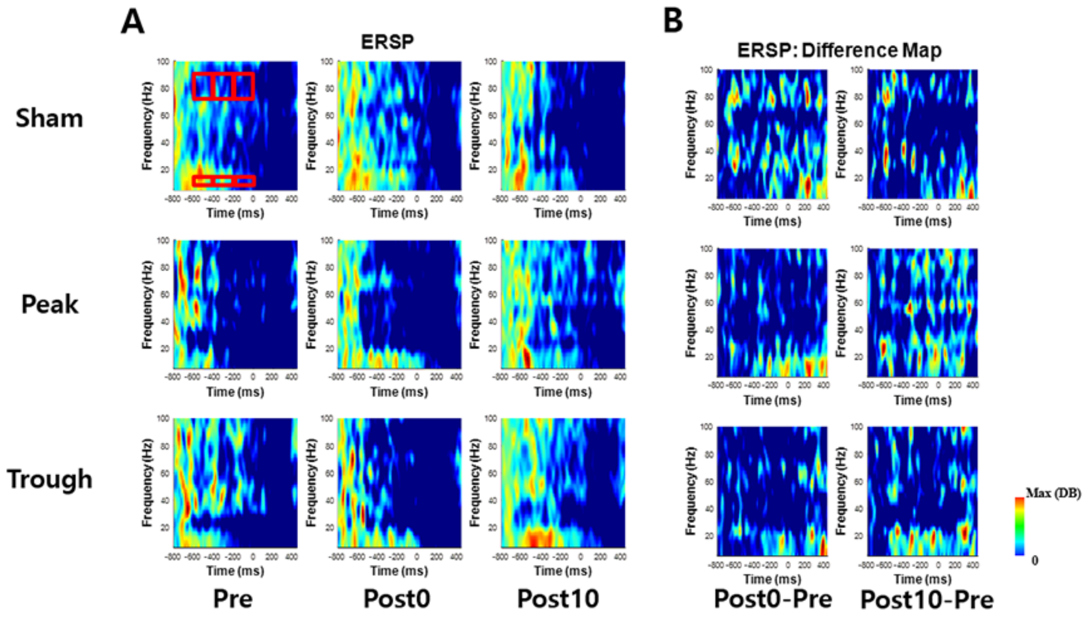

Figure S4. (a) The ERSP during -1000 ms to 500 ms, with time relative to the visual stimulus onset. The red squares indicate the windows of interests, (b) the difference map between sessions, first column represents the difference map of post0-pre, and the second column represents the difference map of post10-pre.

5. Eelctrooculogram (EOG)

The horizontal EOG was measured as the absolute potential difference between two electrodes attached to the outer canthi of both the eyes to estimate the saccadic movements of the eyes during the task. The horizontal EOG was analyzed and compared between each task session under the same stimulation conditions to prove that there was no enforcement of the visual search through saccadic movements. The recorded EOG data were high-pass filtered at 1 Hz, and the signals recorded from electrodes placed over the right eye were subtracted from the signals recorded by the electrode placed over the left eye. The subtracted data were then calibrated by subtracting the mean amplitude of 1 s recordings during fixation prior to each trial onset. Finally, the amplitude of the calibrated data was averaged across trials for each of the same stimulus speed. Since behavioral results showed significant difference only under the stimulation condition of trough for each stimulus sizes, the EOG results during the trough condition is depicted in Fig. S5.

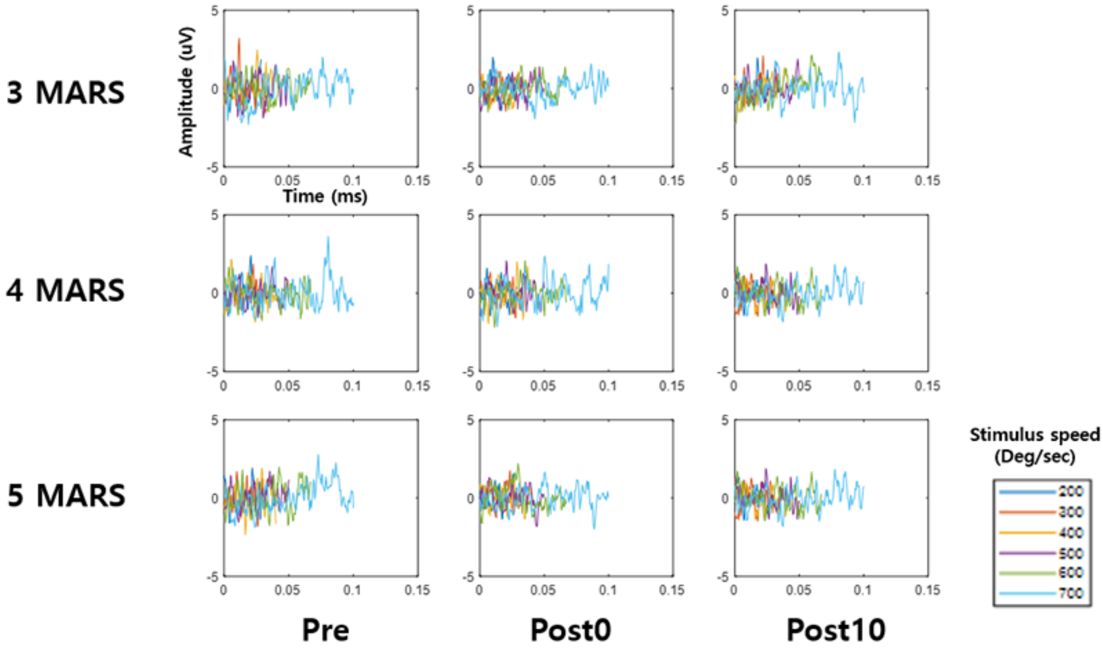

Figure S5. Mean of calibrated EOG amplitude per stimulus size under trough condition. The average EOG amplitude did not exceed 5 uV under any condition, indicating there were no saccadic movements
